# Contributions to our knowledge on avian louse flies (Hippoboscidae: Ornithomyinae) with the first European record of the African species *Ornithoctona laticornis*

**DOI:** 10.1186/s13071-024-06303-8

**Published:** 2024-05-27

**Authors:** Gergő Keve, Tibor Csörgő, Dávid Kováts, Anikó Benke, Attila Tibor Bende, Hunor Ágoston, Attila Mórocz, Ákos Németh, Enikő Anna Tamás, Attila Huber, József Gyurácz, Gábor Keve, Jenő Kontschán, Anna Németh, Sándor Hornok

**Affiliations:** 1 Department of Parasitology and Zoology, University of Veterinary Medicine, Budapest, Hungary; 2 HUN-REN-UVMB Climate Change: New Blood-Sucking Parasites and Vector-Borne Pathogens Research Group, Budapest, Hungary; 3 Department of Genetics, Eötvös Loránd University, Budapest, Hungary; 4 MME BirdLife Hungary, Budapest, Hungary; 5 Hungarian Biodiversity-Research Society, Budapest, Hungary; 6 Fenékpuszta Bird Ringing Station, Fenékpuszta, Hungary; 7 Institute of Wildlife Biology and Management, University of Sopron, Sopron, Hungary; 8 Duna-Dráva National Park Directorate, Pécs, Hungary; 9 Kiskunság National Park Directorate, Kecskemét, Hungary; 10 Kiskunság Bird Protection Association, Izsák, Hungary; 11 Faculty of Water Sciences, LUDOVIKA University of Public Service, Baja, Hungary; 12 Aggtelek National Park Directorate, Jósvafő, Hungary; 13 Department of Biology, Savaria Campus, Eötvös Loránd University, Szombathely, Hungary; 14 Plant Protection Institute, HUN-REN Centre for Agricultural Research, Budapest, Hungary; 15 Department of Plant Sciences, Albert Kázmér Faculty of Agricultural and Food Sciences of Széchenyi, István University in Mosonmagyaróvár, Mosonmagyaróvár, Hungary

**Keywords:** Hippoboscidae, *Ornithoctona laticornis*, *Ornithomya*, Louse fly, Ornithomyinae

## Abstract

**Background:**

Louse flies (Diptera, Hippoboscidae) are important blood-sucking parasites of birds and mammals with a worldwide distribution. The aim of our study was to collect louse flies from birds across multiple sites in Hungary and evaluate the effects of avian traits on louse fly–host relationships.

**Methods:**

Between 2015 and 2022, 237 louse flies were collected from birds at multiple locations in Hungary. The louse flies were identified to species level by morphological and molecular methods. Louse fly species and their seasonal dynamics were analyzed.

**Results:**

Six louse fly species were identified: *Ornithomya avicularia*, *Ornithomya fringillina*, *Ornithomya biloba*, *Ornithomya chloropus*, *Ornithoica turdi* and *Ornithoctona laticornis.* Results of statistical analyses indicated that habitat, migration habits and the feeding places of birds have significant effects on their possible role as hosts of *O. avicularia*, *O. fringillina* and *O. turdi*. Analysis of the temporal distribution of avian louse flies showed different seasonal patterns according to species. Phylogenetic analyses highlighted that *O. turdi* clustered separately from other members of the subfamily Ornithomyinae which thus did not form a monophyletic group.

**Conclusions:**

This study presents one of the longest continuous collections of ornithophilic louse fly species in Europe so far. Avian traits were shown to influence louse-fly infestation. To our best knowledge, this is the first report on *O. laticornis* in Europe. The ability of this African louse fly species to survive in Europe, as demonstrated in the present study, may be an indication of its future establishment*.* Our findings, in accordance with previous reports, also indicated that the subfamily Ornithomyinae should be taxonomically revised.

**Graphical Abstract:**

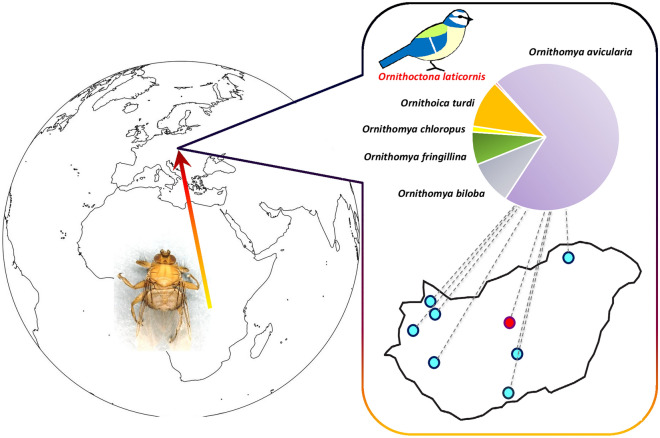

**Supplementary Information:**

The online version contains supplementary material available at 10.1186/s13071-024-06303-8.

## Background

The role of birds in the “transportation” of arthropods with vector potential is long-known [1]. This aspect of bird life as parasite hosts is suspected to become more and more important due to the changes in ecological conditions and which may become a consequence of the currently ongoing climate change [2].

Louse flies (Diptera, Hippoboscidae) are blood-sucking parasites of birds and mammals, with 213 known species worldwide [3]. The family Hippoboscidae contains three subfamilies: Hippoboscinae, Lipopteninae and Ornithomyinae. Ornithophilic louse flies generally belong to the Ornithomyinae subfamily [4]; however, members of the Hippoboscinae subfamily (e.g. *Hippobosca equina* and *Hippobosca longipennis*) can also parasitize birds [5]. Bird lice can negatively affect the health and condition of birds when present and they also play an important role in the ecology of other parasites, possibly contributing to their evolution by phoresis [6]. They can carry a multitude of different pathogens with high veterinary-medical significance, as exemplified by the West-Nile virus [7] and *Babesia* species [8], although their vector role is not yet clear. Research on hippoboscids is flourishing nowadays, i.e. with investigations focusing on their ecology, evolution, and potential role in the transmission of pathogens [3, 9–14].

Although studies on louse flies originating from Central [3] Northern [14], Southern [10], Western [15] and Eastern Europe [16] have also been conducted, only a few of these report long-term evaluations with continuous sample collection. The number of studies conducted on avian ectoparasites steadily increased in Hungary and in other Central European countries during the previous decade [2, 3, 12, 17, 18], but studies on ornithophilic hippoboscids appear to have been neglected compared to other arthropod vectors that are generally considered epidemiologically more important (i.e. ticks and mosquitoes) [19, 20]. Previous Hungarian studies on hippoboscids date back to the previous century and around the turn of the millenium [21, 22]. In light of the above, research on these insects has both regional and international importance.

The aim of the present study was to monitor the louse fly fauna of foraging birds in Hungary, elaborate on the effects of the hosts' attributes (migration habit, habitat, feeding place) on louse fly burden and identify potential trends in the temporal distribution of hippoboscids in Hungary.

## Methods

### Sample collection

**Fig. 1:**
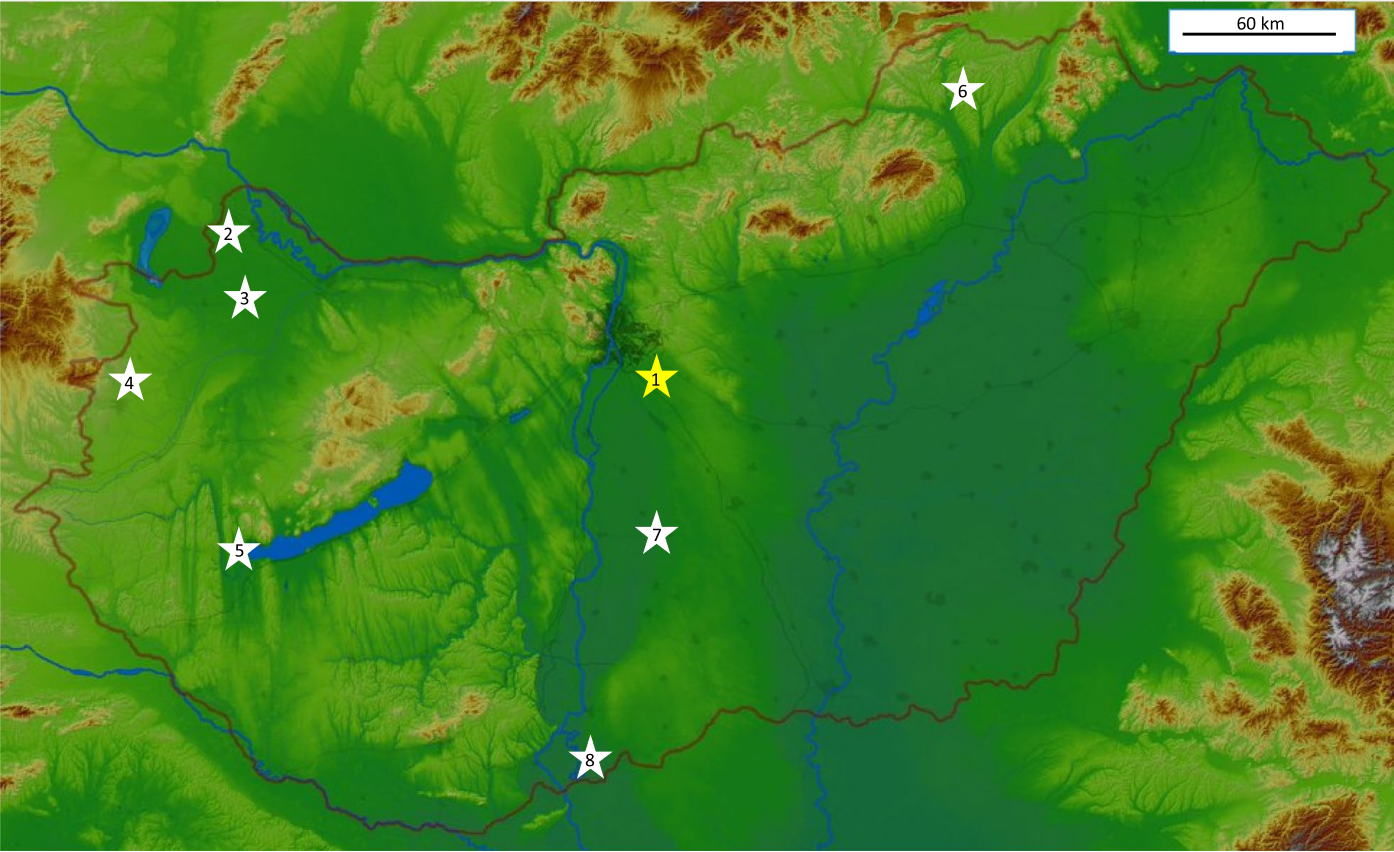
Map of the Carpathian Basin showing sample collection sites (stars with numbers). The yellow star (marked with 1) denotes the ringing station at Ócsa where 8-year-long sampling activities took place. White stars mark other locations where sample collection occurred only in 2022: (2) Lajta-Hanság, (3) Barbacs, (4) Tömörd, (5) Fenékpuszta, (6) Szalonna, (7) Izsák, (8) Dávod

Hippoboscids were collected from passeriform, strigiform and piciform hosts by professional bird-ringers at multiple locations in Hungary (Fig. 1): Ócsa (47°19′ N, 19°13′ E), Fenékpuszta (46°44′N, 17°14′E), Dávod (46°0′N, 18°51′E), Izsák (46°46′N, 19°19′E), Szalonna (48°27′N, 20°42′E) and Tömörd (47°21′N, 16°39′E). Birds were mist-netted for ringing by standard ornithological (Ecotone, Gdynia, Poland) mist-nets (length 12 m, mesh size 16 × 16 mm). During the ringing procedure, birds were examined for the presence of ectoparasites. Samples were also collected at Barbacs (47°38′N, 17°18′E) and Lajta-Hanság (47°50′N, 17°14′E) by licensed hunters during the thinning of Hooded Crow (*Corvus cornix*) population.

Hippoboscids were also caught from the environment of the bird-ringing facilities. As many flies can fall off or escape from the bird during the capturing and the ringing procedure [10], some avian louse flies were found in and around the buildings where the birds were ringed. In these cases, it was impossible to identify the original hosts and, therefore, the term “environment” was used.

In Ócsa, collection activities started in 2015 and continued in all consecutive years until 2022, with collections from March to November. At the other sample collection sites, sample collection started in March 2022 and ended in November 2022.

Ornithophilic louse flies were collected with fine tweezers and stored in 2-ml screwcap tubes filled with 96% ethanol. Data on the collection (date, location, avian host species) were recorded (Additional file 1: Table S1).

### Categorization of birds according to their habitat, migration habit and feeding place

HURING codes were used instead of the birds’ full English and/or binomial names in all figures and tables presented in this study. These abbreviations are explained in Table 1 and Additional file 2: Table S2. English bird species names are capitalized, following international recommendations (https://bou.org.uk/britishlist/bird-names/).

**Table 1:** Total number of ornithophilic louse fly species collected according to the host species

Scientific name of bird species	HURING code for bird species	Total number of infested birds	Louse fly species identified (*n*)					
			*Ornithomya avicularia*	*Onrnithomya biloba*	*Ornithomya fringillina*	*Ornithomya chloropus*	*Ornithoica turdi*	*Ornithoctona laticornis*
*Locustella luscinioides*	LOC LUS	32	36	–	1	–	2	–
*Turdus merula*	TUR MER	25	24	–	–	–	3	–
*Acrocephalus scirpaceus*	ACR SCI	23	21	–	2	–	–	–
*Hirundo rustica*	HIR RUS	21	1	21	–	–	–	–
*Turdus philomelos*	TUR PHI	11	10	–	–	–	3	–
*Acrocephalus arundinaceus*	ACR ARU	9	9	–	–	–	–	–
*Sylvia atricapilla*	SYL ATR	8	4	–	3	–	1	–
*Lanius collurio*	LAN COL	5	–	–	–	–	7	–
*Acrocephalus melanopogon*	ACR MEL	5	4	–	1	–	–	–
*Acrocephalus schoenobaenus*	ACR SCH	5	4	–	1	–	–	–
*Anthus trivialis*	ANT TRI	5	5	–	3	–	–	–
*Emberiza schoeniclus*	EMB SCH	4	2	–	–	–	3	–
*Cyanistes caeruleus*	PAR CAE	4	1	–	–	–	2	1
*Asio otus*	ASI OTU	4	5	–	–	–	–	–
*Erithacus rubecula*	ERI RUB	3	2	–	–	–	1	–
*Corvus cornix*	COR NIX	3	3	–	–	–	–	–
*Strix aluco*	STR ALU	3	7	–	–	–	–	–
*Dendrocopos major*	DEN MAJ	3	5	–	–	–	–	–
*Prunella modularis*	PRU MOD	3	–	–	2	1	–	–
*Emberiza citrinella*	EMB CIT	2	–	–	–	–	2	–
*Panurus biarmicus*	PAN BIA	2	1	–	–	1	–	–
*Luscinia megarhynchos*	LUS MEG	2	2	–	–	–	–	–
*Parus major*	PAR MAJ	2	1	–	1	–	–	–
*Riparia riparia*	RIP RIP	1	–	1	–	–	–	–
*Regulus regulus*	REG REG	1	1	–	–	–	–	–
*Picus viridis*	PIC VIR	1	2	–	–	–	–	–
*Sitta europaea*	SIT EUR	1	1	–	–	–	–	–
*Acrocephalus palustris*	ACR RIS	1	1	–	–	–	–	–
*Passer montanus*	PAS MON	1	1	–	–	–	–	–
*Phylloscopus collybita*	PHY COL	1	–	–	–	–	1	–
*Dendrocopos minor*	DEN MIN	1	1	–	–	–	–	–
*Sylvia communis*	SYL COM	1	–	–	1	–	–	–
Environment	Not applicable	18	14	1	2	1	–	–
Total		193	168	23	17	3	25	1

All louse fly-bird associations can be found in Additional file 1: Table S1. Birds were categorized according to their feeding place, migration habits and habitats according to ornithological data and previous reports [17, 19, 23] as well as the expertise of two of the authors (TCS and DK) (Additional file 2: Table S2). In order to categorize birds according to their feeding places, an “Above ground” category was created. Birds belonging to this group feed on, for example, reed trunks, bushes or branches that do not touch the ground directly but are not far from it either.

### Morphological identification of louse fly species

Louse fly species were identified based on standard taxonomic keys: [3, 15, 24].

### DNA extraction

Seven hippoboscids were chosen for molecular identification: two *Ornithomya avicularia*, and one each from the other louse fly species identified (*Ornithomya fringillina*, *Ornithomya biloba*, *Ornithomya chloropus*, *Ornithoica turdi*, *Ornithoctona laticornis*). The surface of louse flies was disinfected by sequential washing for 15 s in 10% NaClO, tap water and distilled water. For the DNA extraction, one leg of each specimen was cut off. DNA was extracted with the QIAamp DNA Mini Kit (Qiagen, Hilden, Germany) according to the manufacturer's instruction, including an overnight digestion in tissue lysis buffer and Proteinase-K at 56 °C. Extraction controls (tissue lysis buffer) were also processed with the hippoboscid samples to monitor cross-contamination.

### Molecular identification of Hippoboscidae species

The cytochrome *c* oxidase subunit I (*cox* 1) encoding gene was chosen as the target for molecular analysis. The PCR was modified from Folmer et al. [25] and amplifies an ~710-bp-long fragment of the gene. The primers HCO2198 (5′-TAA ACT TCA GGG TGA CCA AAA AAT CA-3′) and LCO1490 (5’-GGT CAACAA ATC ATA AAG ATA TTG G-3’) were used in a reacti on volume of 25 μl, containing 1 U (stock 5 U/μl)HotStarTaq Plus DNA Polymerase (Qiagen, Hilden, Germany), 2.5 μl 10 × CoralLoad Reacti on buffer (including 15 mM MgCl_2_), 0.5 μl PCR nucleotide Mix (Qiagen, Hilden, Germany) (stock 10 mM), 0.5 μl of each primer (stock 50 μM), 15.8 μl ddH_2_O and 5 μl template DNA. For amplification, an initial denaturation step at 95°C for 5 min was followed by 40 cycles of denaturation at 94°C for 40 s, annealing at 48°C for 1 min and extension at 72°C for 1 min. Final extension was performed at 72°C for 10 min.

### Sequencing

A non-template reaction mixture served as the negative control in all PCR analyses. Extraction controls and negative controls remained PCR negative in all tests. The PCR products were purified and sequenced by Eurofins Biomi Ltd. (Gödöllő, Hungary). The BioEdit program was used for quality control and trimming of sequences. The analyses of assembled sequences were performed with BLASTN via GenBank (https://blast.ncbi.nlm.nih.gov). The sequences obtained in the current study were deposited in the GenBank database and are available under accession numbers PP111350–PP111356.

### Statistical analyses

**Fig. 2:**
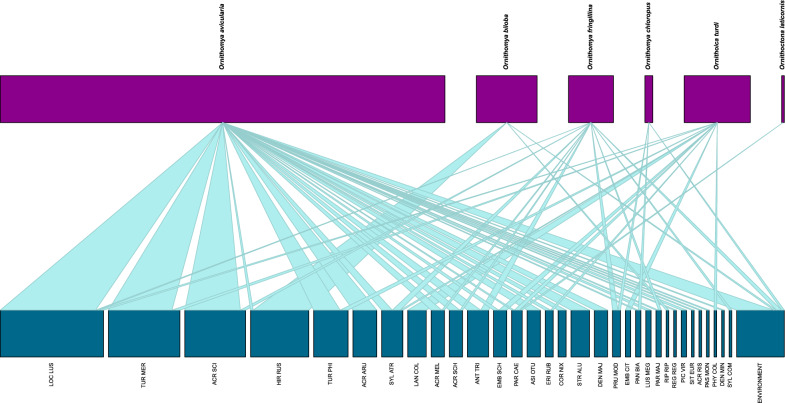
Louse fly–host associations found during the study, visualized on a plotweb. For HURING code, see Table 1 and Additional file 2: Table S2

**Fig. 3:**
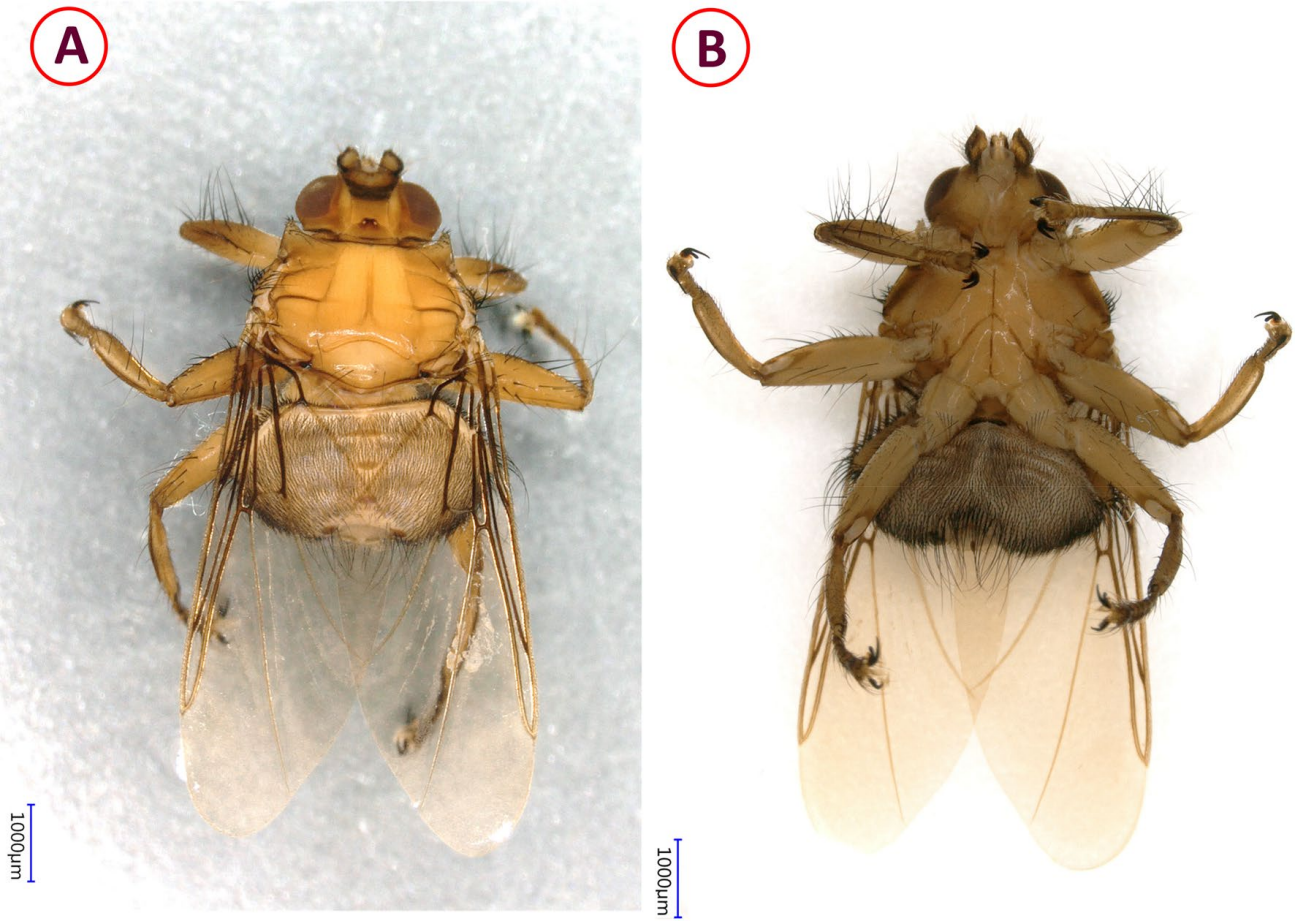
Dorsal (**A**) and ventral (**B**) pictures of the louse fly *Ornithoctona laticornis*

All data from the sample collectors were recorded in Microsoft Office Excel (Microsoft Corp., Redmond, WA, USA), and all calculations, with the except of Fisher’s exact tests and calculations for the phylogenetic tree, were performed in this program. The mean and median rates of infestation were calculated according to Reiczigel et al. [26]. For the comparison of host attributes (habitat, migratory habit, feeding place), Fisher’s exact tests were used (R program 4.3.0) [27]; R program 4.3.0 was also used to generate Fig. 2 (bipartite package) [28]) and Fig. 3 (igraph [29], ggraph [30] and RColorBrewer [31] packages).

### Phylogenetic analysis

In order to construct the tree,* cox*1 nucleotide sequences of hippoboscids were chosen from the GenBank database (https://www.ncbi.nlm.nih.gov/nucleotide/). Sequences were aligned with MUSCLE (MEGA 11 program [32]). To root the tree, we chose three species from the Calyptratae subsection.

The evolutionary history was inferred by using the maximum likelihood method and general time reversible (GTR) model [33]. The percentage of trees in which the associated taxa clustered together is shown below the branches. The tree is drawn to scale, with branch lengths measured in the number of substitutions per site. This analysis involved 46 nucleotide sequences. There were a total of 589 positions in the final dataset. Evolutionary analyses were conducted in MEGA 11 [32].

## Results

### Species and numbers of louse flies

During the 8-year-long sample collection period, 237 louse flies were collected from 175 birds (*n*_flies_ = 219) of 32 species, and from the environment of the ringing facilities (*n*_flies_ = 18) at multiple locations across Hungary. Mean and median intensity of infestation was 1.13 and 1, respectively. According to the morphological identification, the louse flies found belonged to six species: *Ornithomya avicularia (n* = 168*)*, *Ornithomya biloba* (*n* = 23), *Ornithomya fringillina* (*n* = 17), *Ornithomya chloropus* (*n* = 3), *Ornithoica turdi* (*n* = 24) and *Ornithoctona laticornis* (*n* = 1)*.* The parasite–host associations are visualized in Fig. 2 and listed in Table 1.

**Fig. 4:**
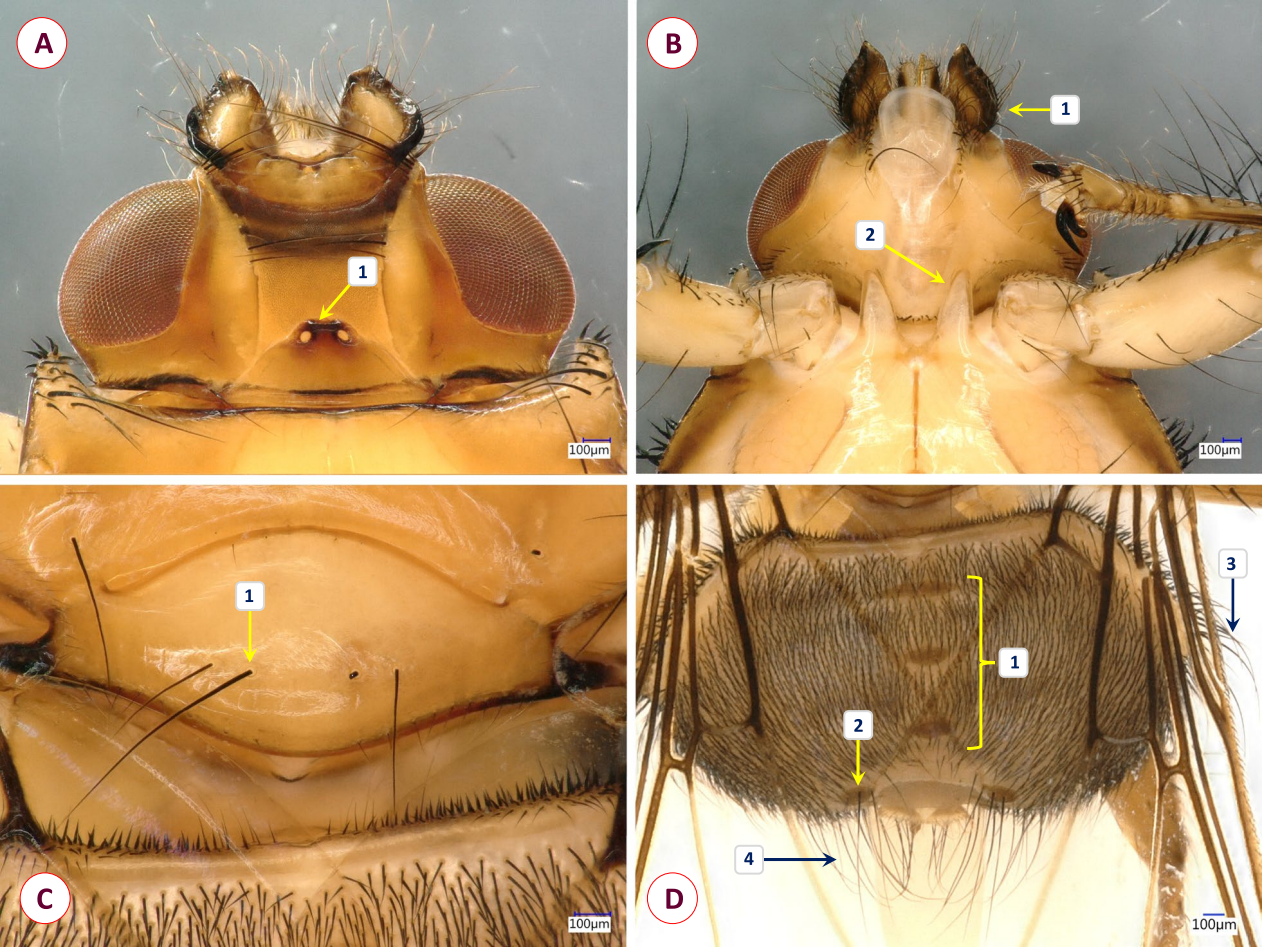
Key morphological characters of the louse fly *Ornithoctona laticornis*. **A** Dorsal view of the head (1 denotes the anterior ocellus situated slightly above the level of posterior eye margins). **B** Ventral view of the head, mesosternal processes (1 denotes the antennae, which were twice as long as broad; 2, the length of the mesosternal process). **C** Scutellum with four prominent hairs (1 denotes the medial hairs, which were twice as long as the lateral ones), **D** Dorsal view of the abdomen (1 denotes three median tergal plates; 2, two log hairs on both plates of tergite six; 3, antero-lateral area of abdomen with long hair; 4, caudal area of abdomen with long hair)

**Fig. 5:**
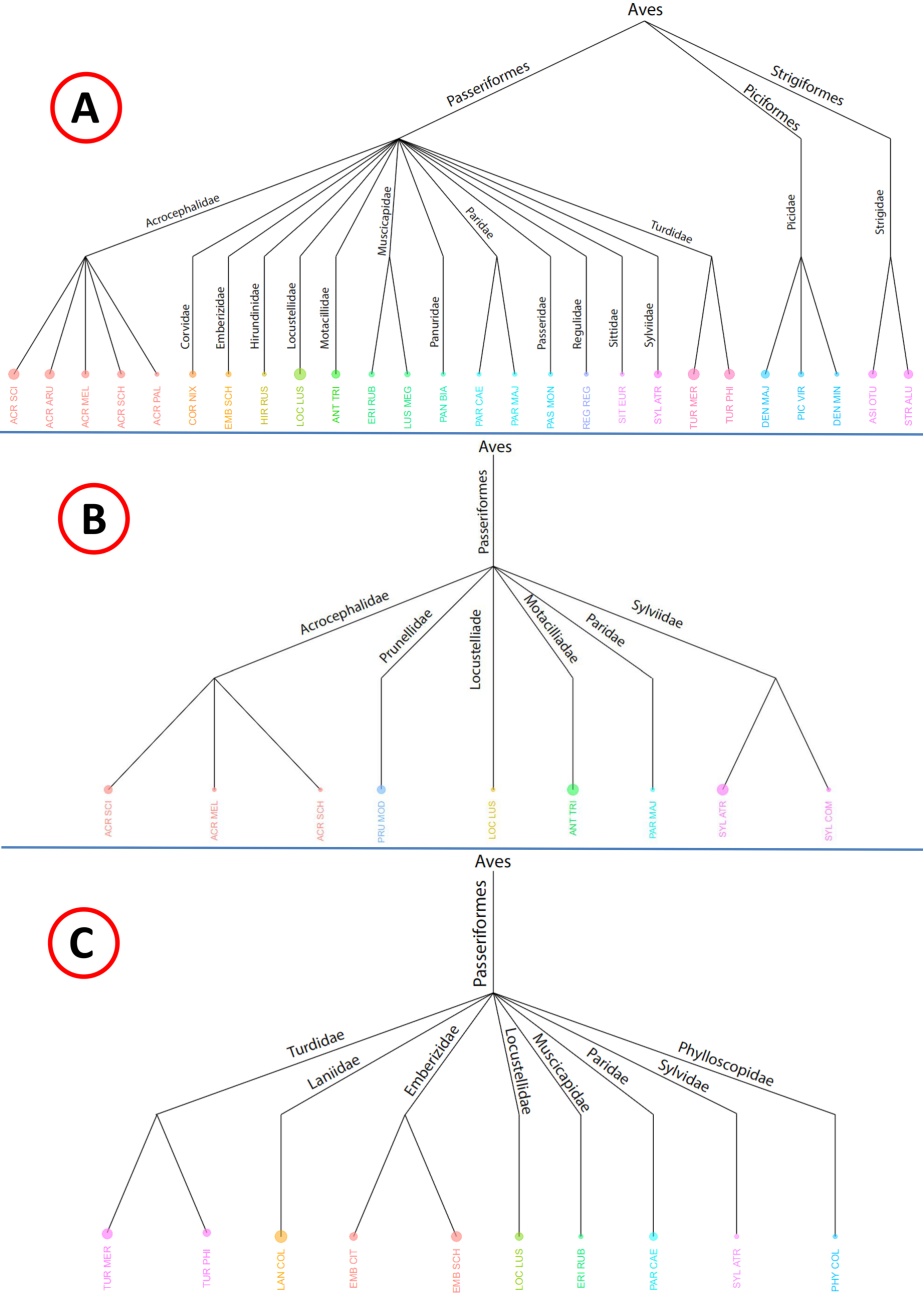
Taxonomy of the host species of the louse flies *Ornithomya avicularia* (**A**), *Ornithomya fringillina* (**B**) and *Ornithoica turdi* (**C**) visualized on dendrograms

**Fig. 6:**
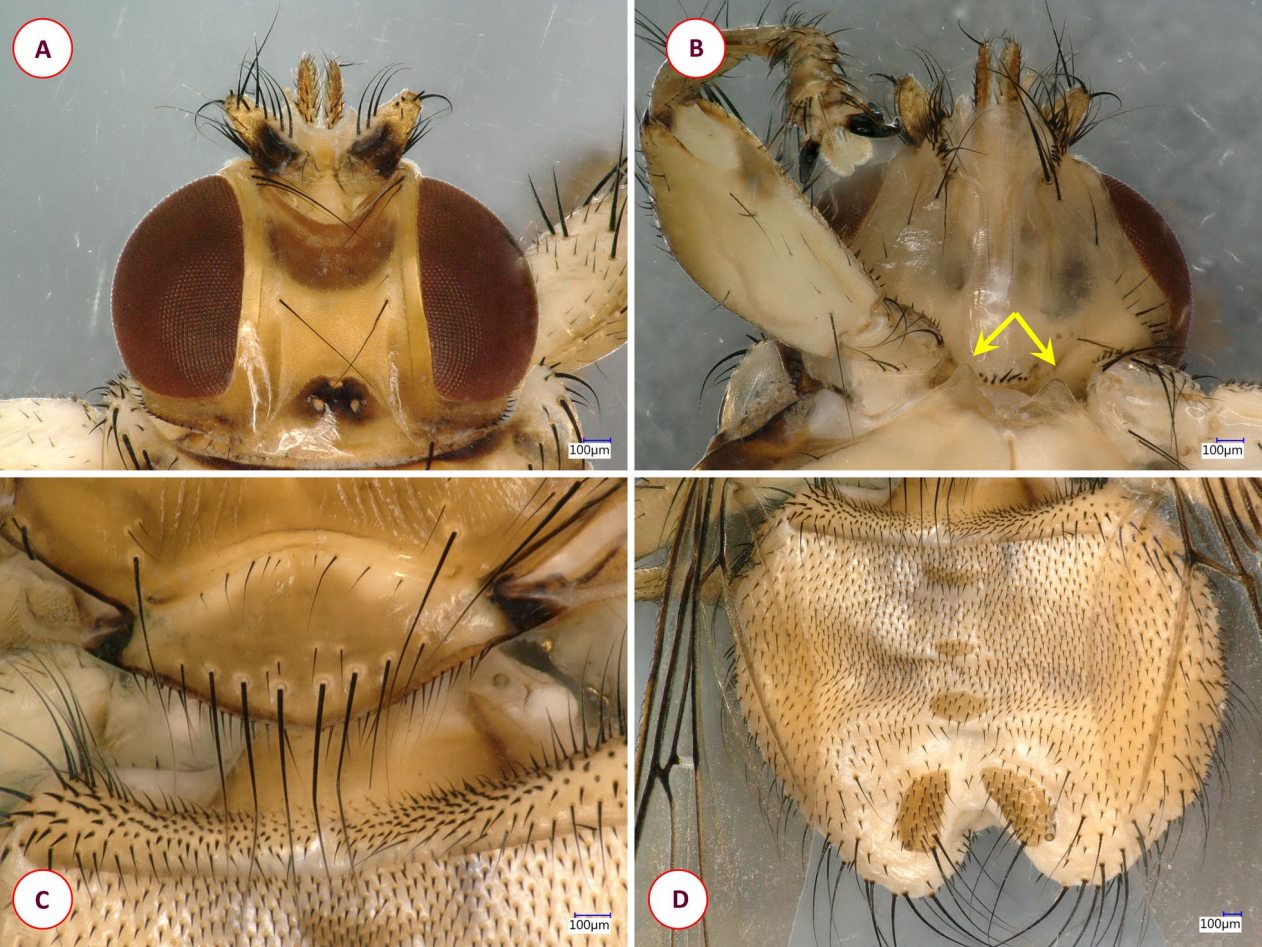
Key morphological characters of the louse fly *Ornithomya avicularia*. **A** Dorsal view of the head. **B** Ventral view of the head, mesosternal processes (arrows). **C** Scutellum. **D** dorsal view of the abdomen

**Fig. 7:**
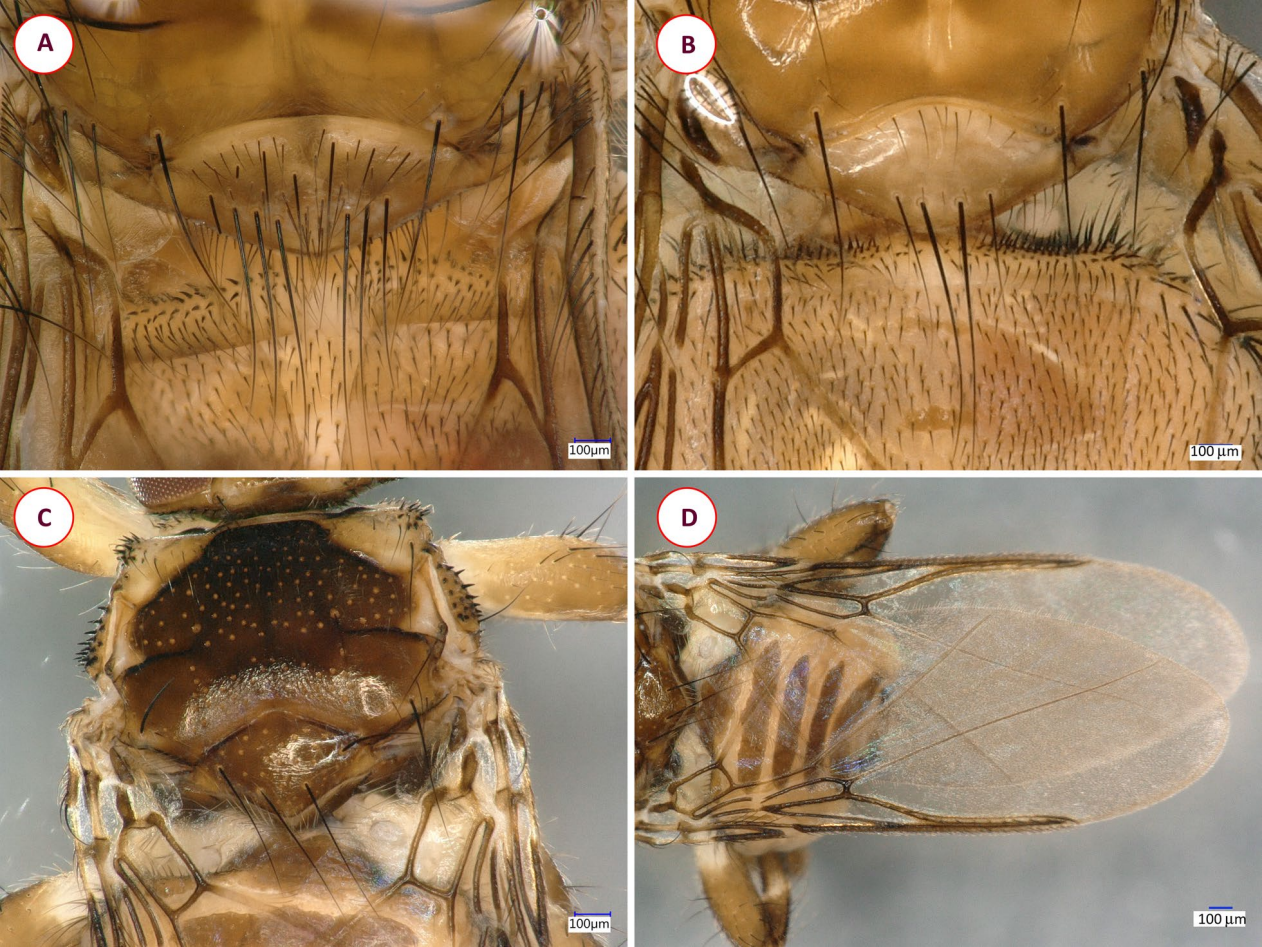
The scutellum of the louse flies *Ornithomya biloba* (**A**) and *Ornithomya fringillina* (**B**). **C**, **D** Key morphological characters of *Ornithoica turdi*: **A** dorsal view of the thorax, scutellum, **D** wings

**Fig. 8:**
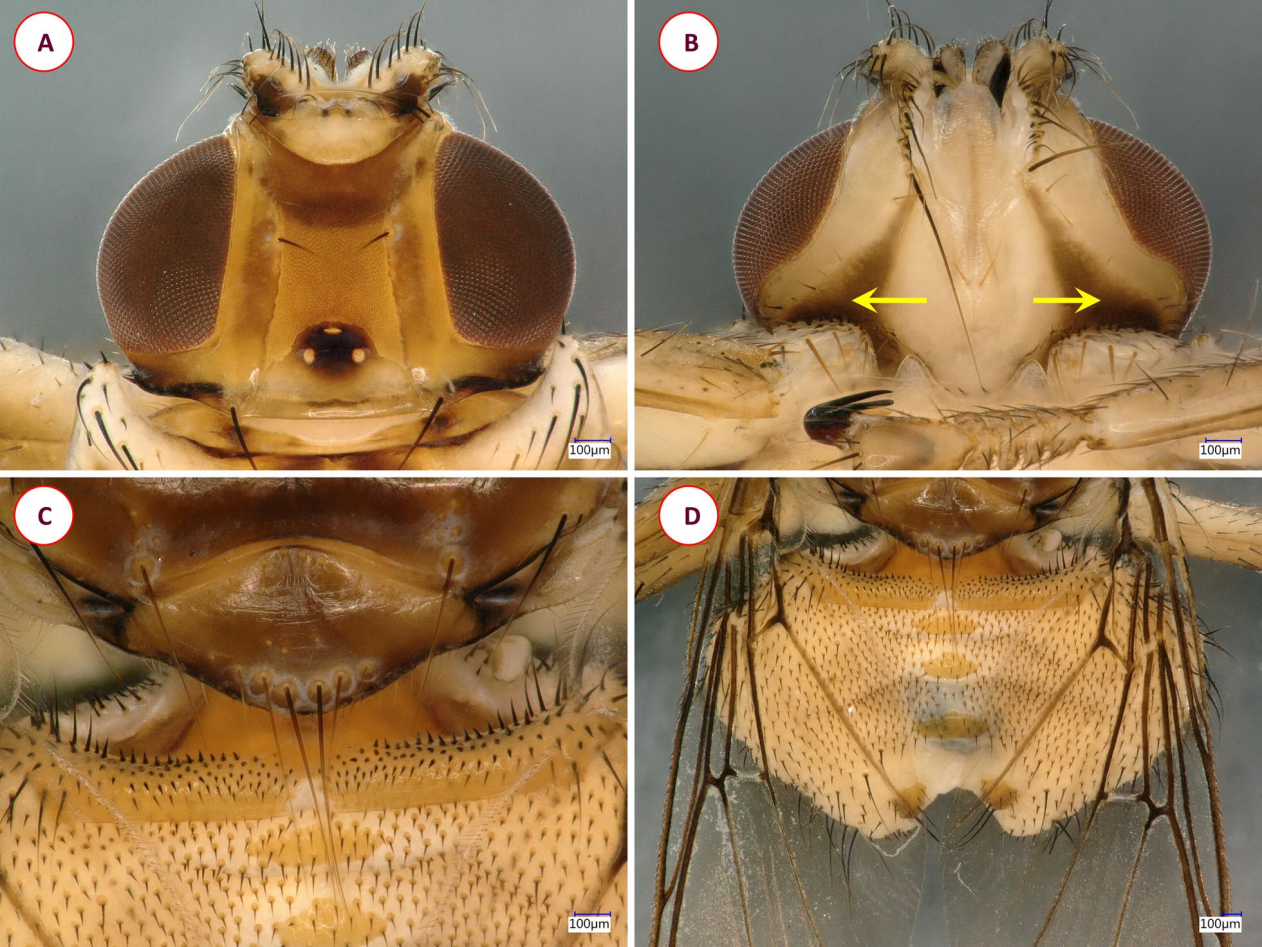
Key morphological characters of the louse fly* Ornithomya chloropus*. **A** Dorsal view of the head. **B** Ventral view of the head showing sharp, triangular brown spots (arrows). **C** Scutellum. **D** Dorsal view of the abdomen

The maximum number of different louse fly species infesting the same bird was two. This only happened for two birds: a Song Thrush (*Turdus philomelos*) and a Common Reed Bunting (*Emberiza schoeniclus*). The co-infestation detected on these birds consisted of *O. avicularia* and *O. turdi* (one–one specimens) (Additional file 1: Table S1). The species and number of louse flies identified and their bird hosts are as follows:

- *Ornithoctona laticornis* (*n* = 1). Only one specimen of this species was found. It was removed from a Eurasian Blue Tit (*Cyanistes caeruleus*) (Table 1; Figs. 3, 4; Additional file 1: Table S1).
- *Ornithomya aviculara* (*n* = 168). This species of louse fly was the most abundant during the study period, with 71% of the detected louse flies belonging to this species. The most common host of this species was Savi’s Warbler (*Locustella luscinioides*) (*n*_birds_ = 29,* n*_flies_ = 36) (Table 1). *Ornithomya avicularia* specimens were collected from 26 different bird species of three orders (Passeriformes, Strigiformes, Piciformes) (Figs. 5, 6).
- *Ornithomya biloba* (*n* = 23). Specimens of this species were collected from Barn Swallow (*Hirundo rustica*) (*n*_birds_ = 20, *n*_flies_ = 21) and from Sand Martin (*Riparia riparia*) (*n*_birds_ = 1, *n*_flies_ = 1); one from the environment (Table 1; Fig. 7).
- *Ornithomya fringillina* (*n* = 17). Fifteen specimens of this louse fly species were removed from nine different passeriform bird species among which the most abundant were the Eurasian Blackcap (*Sylvia atricapilla*) (*n*_birds_ = 3, *n*_flies_ = 3) and the Tree Pipit (*Anthus trivialis*) (*n*_birds_ = 3, *n*_flies_ = 3). Two specimens were collected from the environment (Table 1; Figs. 5, 7).
- *Ornithomya chloropus* (*n* = 3). In total, three specimens of this louse fly species were collected: one from a Dunnock (*Prunella modularis*) (*n*_bird_ = 1, *n*_fly_ = 1), one from a Bearded Reedling (*Panurus biarmicus)* (*n*_bird_ = 1, *n*_fly_ = 1) and one from the environment (Table 1, Fig. 8).
- *Ornithoica turdi* (*n* = 25). This louse fly species was the second most abundant species, even though they accounted for only 10.5% of all louse flies. The most common host of this species was the Red-backed Shrike (*Lanius collurio*) (*n*_birds_ = 5, *n*_flies_ = 7). *Ornithoica turdi* were found feeding on 10 different bird species during the study period (Table 1; Figs. 5, 7).

### Molecular identification and phylogenetic analyses

In general, based on the* cox*1 gene of the representative louse fly specimens, the identity of all six louse fly species was confirmed (*O. avicularia*, *n* = 2 specimens tested; *O. biloba,* *n* = 1; *O. fringillina,* *n* = 1; *O. chloropus*, *n* = 1; *O. turdi*, *n* = 1; *O. laticornis*, *n* = 1).

**Fig. 9:**
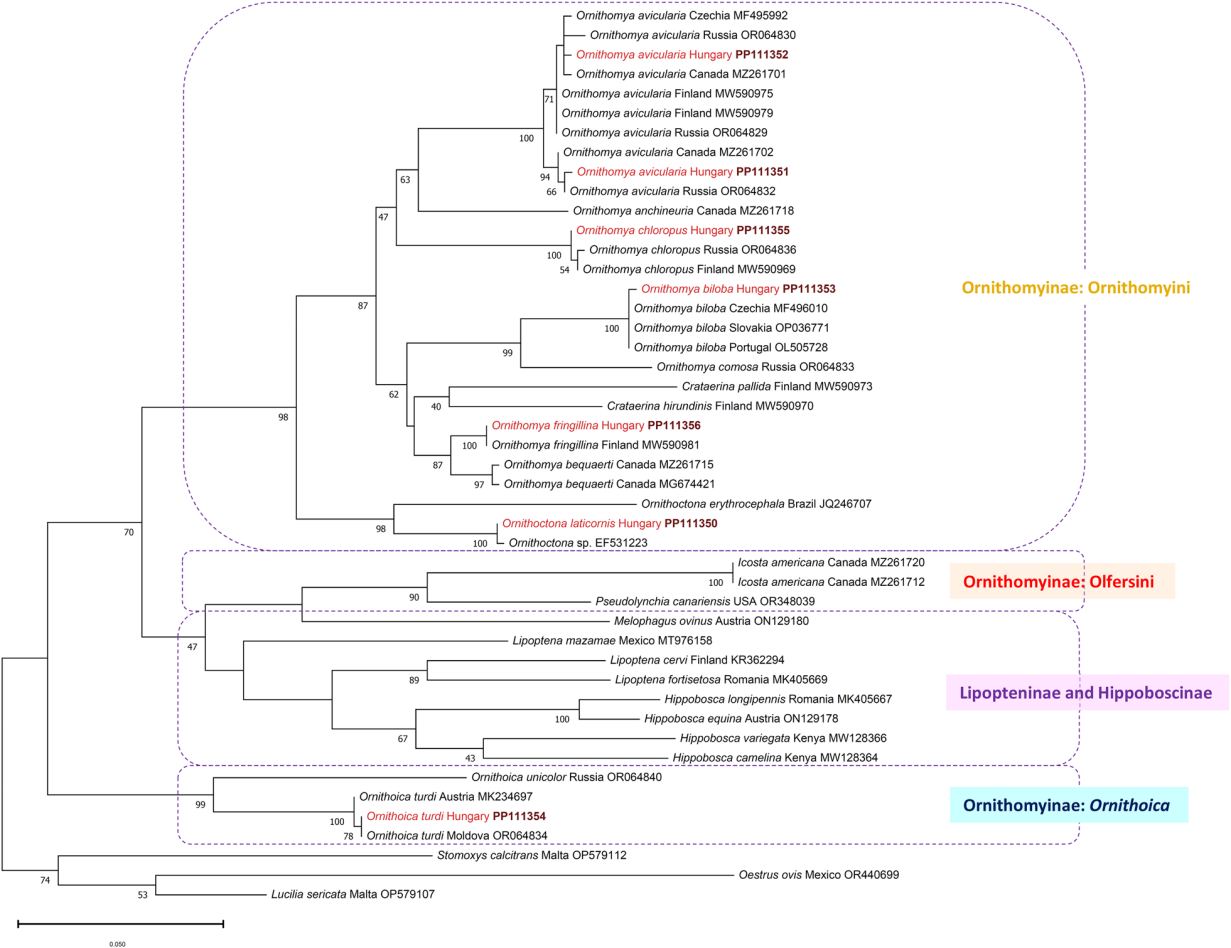
Phylogenetic tree based on the cytochrome *c* oxidase subunit I (*cox*1) gene of hippoboscid flies. The evolutionary history was inferred using the maximum likelihood method and general time reversible (GTR) model

More specifically, according to their* cox*1 sequences:

- *Ornithoctona laticornis* (PP111350) showed 99.83% identity to an *Ornithoctona* sp. (EF531223) from the collection of the North Carolina State University (host and collection site are unknown).
- The two *O. avicularia* specimens (PP111351 and PP111352) belonged to different haplotypes (98.28% identity). PP111351 showed 99.69% identity to *O. avicularia* (OR064832) from Russia and 99.53% to *O. avicularia* (MZ261702) from Canada. PP111352 showed 99.69% identity to *O. avicularia* (OP035933) from Slovakia and the same percent of identity to multiple *O. avicularia* specimens from Canada (MZ261701, MZ261708, MZ261714) (Fig. 9).
- *Ornithomya biloba* (PP111353) was 99.84% identical to *O. biloba* specimens from the Czech Republic (MF496010), Slovakia (OP036771) and from Portugal (OL505728) (Fig. 9).
- *Ornithoica turdi* (PP111354) showed 100% identity to *O. turdi* (OR064834) from Moldova, and 99.69% to *O. turdi* (MK234697) from Austria (Fig. 9).
- *Ornithomya chloropus* (PP111355) showed 99.84% identity to *O. chloropus* specimens from Finland (MW590960, MW590969) and from Russia (OR054225) (Fig. 9).
- *Ornithomya fringillina* (PP111356) showed 100% identity to *O. fringillina* specimens from Finland (MW590981, MW590974, MW590963) (Fig. 9).

### Statistical analyses

Due to the low numbers of *O. chloropus* and *O. laticornis*, no statistical probes were performed involving these species. *Ornithomya biloba* was also excluded from the analysis as its host specificity towards the Hirundinidae family would have led to biased (or obvious) results.

According to our analyses, there were significant differences between the host habitats (reed, forest, meadow, forest/meadow) of *O. avicularia* and *O. turdi* (*P* < 0.0001). *Ornithomya avicularia* was the most abundant louse fly found on reed-associated birds, and *O. turdi* was the most commonly found louse fly on forest- and meadow-associated hosts. In the case of *O. avicularia* and *O. fringillina*, the result of the same comparison was non-significant (*P* = 0.2958). The difference between *O. fringillina* and *O. turdi* was also non-significant (*P* = 0.1985) (Table 2).

**Table 2:** Number of louse fly species according to the habitat of their hosts

Louse fly species	Reed-associated birds (R)	Meadow-associated birds (M)	Forest-associated birds (F)	F/M
*Ornithomya avicularia*	78	5	35	36
*Ornithomya fringillina*	5	1	6	3
*Ornithoica turdi*	5	9	8	3

Comparisons were made based on the migration habits (resident, resident/short-distance migrants, short-distance migrants and long-distance migrants) of the hosts of *O. avicularia, O. fringillina* and *O. turdi.* The difference between the hosts of *O. avicularia* and *O. fringillina* was significant (*P* = 0.0036) as we found no *O. fringillina* on short-distance migrants; however the difference was non-significant in the comparison of the hosts of *O. avicularia* and *O. turdi* (*P* = 0.4033). A significant difference was also visible for the hosts of *O. fringillina* and *O. turdi,* as the latter was frequently found on short-distance migrants (*P* = 0.0343) (Table 3).

**Table 3:** Number of louse fly species according to the migration habit of their hosts

Louse fly species	Resident birds (R)	Short-distance migrant (SDM)	R/SDM	Long-distance migrant
*Ornithomya avicularia*	10	47	18	79
*Ornithomya fringillina*	1	0	6	8
*Ornithoica turdi*	2	9	5	9

The avian hosts were also categorized according to their feeding place (Ground, Above ground, Ground/Above ground). There was a significant difference between the hosts of *O. avicularia* and *O. fringillina* (*P* = 0.0024) as *O. fringillina* was not found feeding on birds belonging to the “Ground” category during the study period; in contrast, 36% of *O. avicularia*-parasitized birds belonged to the “Ground” category. The difference was also significant for the hosts of *O. avicularia* and *O. turdi* (*P* = 0.0068), with 64% of the *O. turdi* specimens found on birds belonging to the “Ground” category of feeding. The difference between *O. fringillina* and *O. turdi* according to feeding place was also significant (*P* < 0.0001) (Table 4).

**Table 4:** Number of louse fly species according to the feeding places of their hosts

Louse fly species	Ground	Above ground	Ground/Above ground
*Ornithomya avicularia*	48	93	12
*Ornithomya fringillina*	0	11	4
*Ornithoica turdi*	16	9	0

### Temporal distribution of louse flies

**Fig. 10:**
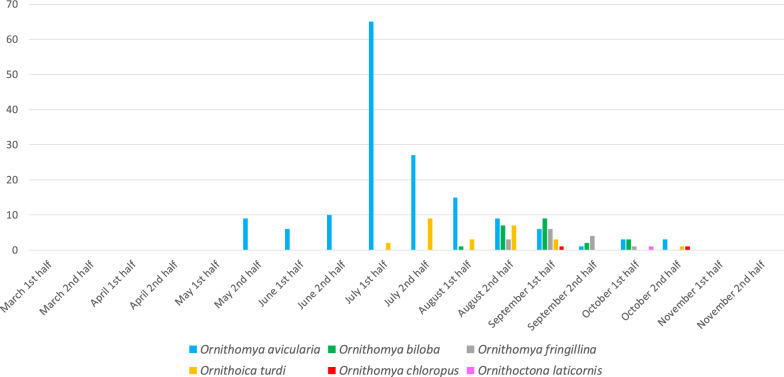
Temporal distribution of the louse fly species found on birds during the 8-year-long study period

The temporal distribution of louse fly species found on birds during the 8-year-long study period is visualized in Fig. 10. It should be noted that in this study no sample collection was conducted during the winter. *Ornithomya avicularia* specimens were recovered from the second half of May up to the end of October, reaching their peak abundance in the first half of July. *Ornithomya biloba* specimens were only collected from the first half of August until the first half of October and were most common in the first half of September, even though its most common hosts (*Hirundo rustica*) were occasionally caught and checked for the presence of louse flies during the springtime. The occurrence of *O. fringillina* was at its highest in the first half of September; this species was present from the second half of August until the first half of October. *Ornithoica turdi* showed activity from the first half of July until the second half of October, with peak abundance in the second half of July. Only three specimens of *O. chloropus* were collected: one on 5 September 2022 and one on 31 October 2020; for the third specimen, the precise date of collection was not recorded, but is estimated to be October 2019 (Additional file 1: Table S1). *Ornithoctona laticornis* was collected only once, on 9 October 2016.

## Discussion

Six bird-associated louse fly species were identified and analyzed. For several species, we report here for the first time molecular and host-related data from either Central European or European countries. Therefore, our results supplement missing information on the ecology and phenology of relevant louse fly species in this geographical region. We also addressed host-related factors of louse-fly infestation and the taxonomic uniformity of this group.

### Identified louse fly species

To our best knowledge, this is the first time that *O. laticornis* has been found in Europe. In 1984, Hutson [15] suggested that the appearance of this louse fly species in Europe could be expected, since it had been found on multiple Palearctic migrants in Africa. However, up to the present study, it had only been reported in Central and South Africa [34] and Madagascar [24]. Interestingly enough, our specimen was found feeding on a Blue Tit on 9 October 2016, which is long after the spring migration period. Not much is known about the life-cycle of *O. laticornis*, but other hippoboscid adults can survive for around 4 months, and the duration of the pupal stage varies from 19 to 23 days in the summer and from 20 to 36 days in the winter [35]. Also, bird-specific louse flies tend to overwinter in pupal form in Europe, which may indicate even longer pupal stages [21]. The pupae of hippoboscids (in general) usually can be found in bird nests, on the hair of mammalian hosts or on the ground [3]. Based on this information, one hypothesis is that a bird carried an imago from Africa during the spring migration and that the adult fly survived until October in Hungary. However, due to the relatively large temporal distance (approx. 5–7 months) between the spring migration of birds (March–May) and the finding of the *O. laticornis* specimen, it is also conceivable that an already fertilized female *O. laticornis* had arrived in Hungary on a migrating bird in the spring and had been able to lay a larva ready to pupate that later hatched. In the latter scenario, the imago was probably second-generation. A second hypothesis is suggested from the work of Hutson [15]: *Ornithoctona laticornis* can occasionally be found in Europe, but due to its close morphological resemblance to *Ornithomya avicularia* (Figs. 4, 6) some specimens may have been misidentified in the past. According to European keys, the morphological similarity between *O. laticornis* and *Ornithomya rupes* may be even more deceiving [3]. Therefore, population(s) of *O. laticornis* may be currently present in Europe. At this time we do not have enough information to draw accurate conclusions. However, regardless of how this specimen came to Hungary, it was found on a resident bird, which shows that *O. laticornis* can survive Central European conditions, not just in the summer, but in the autumn. This finding highlights the importance of comprehensive research on wild bird parasites as due to the migratory nature of their hosts, these animals can be indicators of the direct effects of climate change.

The other louse fly species identified in this study as *Ornithomya avicularia*,* O. fringillina*,* O. biloba*,* O. chloropus* and* Ornithoica turdi* have all been previously reported in Hungary [22]. The euryxenous natures of *O. avicularia*, *O. turdi* and *O. fringillina* have long been known, as has the host specificity of *O. biloba* towards the Hirundinidae family (especially the Barn Swallow) [10, 13, 16, 22, 36, 37].

*Ornithomya avicularia* was the most abundant louse fly in the present study, the second most abundant was *O. turdi*, followed by *O. biloba*, *O. fringillina* and *O. chloropus*. A similar study recently performed in another Central European country, the Czech Republic, reported different relative abundancies [13]; for example, *O. biloba* and other stenoxenous species were represented in much larger numbers. The main reason for this difference is that in the study from the Czech Republic, nestlings of Barn Swallows and Swifts (*Apus apus*) were also checked for potential louse flies. Therefore, not only were an enormous number of *O. biloba* specimens collected but other stenoxenous louse flies were also found in the nests of the birds, namely *Crataerina hirundinis* and *Crataerina pallida*. In our study, no bird nests were examined and Barn Swallows were only occasionally caught, which explains the relatively low number of *O. biloba,* as due to the random nature of the sample collection, the relative presence of *O. biloba* (and other host-specific species) is also highly affected by the relative number of swallows among the examined hosts.

### Molecular identification and phylogenetic analysis

We report here the nucleotide sequence of *O. laticornis* (PP111350) for the first time. Although our sequence showed 99.83% identity to an *Ornithoctona* sp. (EF531223) [38], the host and site of origin of EF531223 are unknown (from the collection of the North Carolina State University). Base on the* cox*1 gene, the closest relatives (that are available in the GenBank database) of our *O. laticornis* (PP111350) specimen are this *Ornithoctona* sp. from the North Carolina State University collection (EF531223) and *Ornithoctona erythrocephala* from Brazil (JQ246707) [39] (Fig. 9).

Based on the* cox*1 gene, the genus *Ornithoica* seems to show a more distant genetic relationship to genera belonging to the subfamily Ornithomyinae (*Ornithomya*, *Ornithoctona*, *Icosta*, *Pseudolynchia*, *Crataerina*) than it does to certain representatives of the subfamilies Hippoboscinae (*Hippobosca equina*, *H. longipennis, H. variegata*) and Lipopteninae (*Lipoptena mazamae*, *L. cervi*, *L. fortisetosa* and *Melophagus ovinus*) (Fig. 9). This is in line with a recent report from Russia [40]. As shown in a previous study, [39] this genetic relationship also holds when the phylogenetic analysis is based on 18S ribosomal DNA. These results suggest that the genus *Ornithoica* might belong to a different subfamily, and that the taxonomy of Ornithomyinae should be revised.

### Statistical analyses

According to our statistical analyses, the migration habit, the habitat type and the feeding habit of birds affect their potential as louse fly hosts in Hungary. In our study, three euryxenous louse fly species were examined in this regard and significant differences were found between the migration habits of the hosts of *O. avicularia* and *O. fringillina.* In a previous study from the Czech Republic, significant differences were found between the migration habits of the hosts of *O. avicularia* and *O. fringillina* as well; however, in that study *O. avicularia* was far less common on long-distance migrant birds than on short-distance migrants [13]. In contrast, a study from Finland found no significant differences between the migration habits of the hosts [14]. These results might suggest that host preference patterns may differ under different climates, or that the migratory habit alone cannot explain host specificity patterns, but other factors (e.g. ornithological and geographic) may influence the results as well. Nevertheless, both species were the most common on long-distance migrants, and only differed by their ratios. The difference between the hosts of *O. avicularia* and *O. turdi* in the same context was not significant; however, the difference between the hosts *O. turdi* and *O. fringillina* was significant. The latter significance may strengthen the results of the previous two tests.

The habitat-association of hosts also seemed to be different in the case of *O. avicularia* and *O. turdi* (*P* < 0.0001), as *O. avicularia* was the most common on reed-associated birds, and *O. turdi* was the most abundant on forest- and meadow-associated birds. The difference was less pronounced and non-significant between *O. avicularia* and *O. fringillina*, and also non-significant between *O. fringillina* and *O. turdi*. In contrast to this, both *O. avicularia* and *O. fringillina* preferred birds with a forest habitat, but were uncommon on birds from “wetlands” in Finland [14].

Birds were categorized according to their feeding place as well. Interestingly, each comparison demonstrated significant differences (*O. turdi *vs* O. avicularia*, *O. avicularia* vs* O. fringillina* and *O. fringillina * vs* O. turdi*). These results show that despite all three of the hippoboscid species that were statistically examined being able to develop wings and to fly [15, 40], their host selection was influenced by the hosts feeding height. Specifically, *O. turdi* predominated on birds feeding at ground level, whereas *O. fringillina* was absent on birds exclusively feeding at ground level. *Ornithomya avicularia* was approximately twice as abundant on birds feeding above ground compared to those feeding on the ground (Table 4).

### Temporal distribution of louse fly species

In the present study, most of the louse fly species (*O. turdi, O. fringillina, O. chloropus)* were the most active at the end of the summer and in the autumn. *Ornithomya biloba* flies were only collected during the autumn migration of their hosts (Barn Swallow, and other species of Hirundinidae). During the spring migration and the roosting season, none of the previously mentioned louse fly species were found, despite the fact that in other countries *O. biloba* has been found on early swallows [41]. In terms of temporal distribution, the appearance of *O. avicularia* preceded all other louse flies, as this species was active from the second half of May and remained active during the sample collection period. This means that this species was the only one that was found to be active on foraging birds during the main nesting season (Fig. 10). It is important to note that our sample collection started in March and ended in November each year. Although this interval includes the spring (March–May) and autumn migration (September–November) periods [42], only randomly caught birds were examined for the presence of louse flies and no bird nests were checked during the study. Therefore, these results are only relevant to the activity of flies on flying and/or foraging birds, except the wintering period.

## Conclusions

This is the first report of *O. laticornis* in Europe, as well as the first molecular-phylogenetic analysis of this louse fly species. In accordance with previous studies, the migration habit, the habitat type and the feeding habits of birds were found to affect their potential role as the hosts of *O. avicularia*, *O. fringillina* and *O. turdi,* but these patterns may vary in different geographical regions. According to our analyses and data from available literature, members of the genus *Ornithoica* show distant phylogenetic clustering to genera belonging to the subfamily Ornithomyinae (where it was hitherto assigned), necessitating taxonomic revision of this group in the near future.

### Supplementary Information

**Additional file 1: Table S1. **All individual findings of the study.

**Additional file 2 Table S2. **Categorization of the bird species found to be louse fly infested during the study, according to their migration habit, habitat and feeding place.

## Data Availability

The sequences obtained in the current study were deposited in the GenBank database and are available under accession numbers PP111350–PP111356 (https://www.ncbi.nlm.nih.gov/genbank/). All other relevant data are included in the manuscript and its appendices.
