# Supplementary material for: Contributions to our knowledge on avian louse flies (Hippoboscidae: Ornithomyinae) with the first European record of the African species Ornithoctona laticornis
Source: Parasit Vectors. 2024 May 27;17:237. doi: 10.1186/s13071-024-06303-8 (PMC11129389; doi:10.1186/s13071-024-06303-8)
Supplement: Supplementary file 1 — Additional file 1: Table S1. All individual findings of the study. [file 13071_2024_6303_MOESM1_ESM.pdf]

Table S1: All individual findings of the study

ID	Bird Species	Date	Place	*Ornithomya avicularia*	*Onrnithomya biloba*	*Ornithomya fringillina*	*Ornithomya chloropus*	*Ornithoica turdi*	*Ornithoctona laticornis*
KG64	TUR MER	2022.05.28	Ócsa	1					
KG91	ACR SCI	2022.07.10	Ócsa	1					
KG92	LOC LUS	2022.07.11	Ócsa	1					
KG106	ACR ARU	2022.07.16	Ócsa	1					
KG107	ACR SCI	2022.07.16	Ócsa	1					
KG108	ACR SCI	2022.07.16	Ócsa	1					
KG115	LOC LUS	2022.07.23	Ócsa	1					
KG119	LOC LUS	2022.07.30	Ócsa					1	
KG120	LAN COL	2022.07.31	Ócsa					1	
KG123	TUR PHI	2022.08.06	Ócsa					1	
KG146	EMB SCH	2022.08.23	Ócsa	1				1	
KG147	ERI RUB	2022.08.23	Ócsa					1	
KG149	ACR SCI	2022.08.24	Ócsa	1					
KG150	EMB CIT	2022.08.24	Ócsa					1	
KG151	HIR RUS	2022.08.24	Ócsa		1				
KG172	PAR CAE	2022.09.06	Ócsa					1	
SO9	COR NIX	2022.07.11	Barbacs	1					
SO14	COR NIX	2022.08.08	Lajta-Hanság	1					
SO15	COR NIX	2022.08.10	Lajta-Hanság	1					
NA9	ACR MEL	2022.07.13	Izsák	1					
NA20	LOC LUS	2022.07.15	Izsák	1					
NA22	PAN BIA	2022.07.15	Izsák	1					
NA29	LOC LUS	2022.07.17	Izsák	1					
NA48	ACR MEL	2022.07.19	Izsák	1					
NA68	ACR ARU	2022.08.11	Izsák	1					
NA73	ACR SCI	2022.08.28	Izsák			1			
BA16	LOC LUS	2022.06.26	Dávod	1					
HA29	EMB CIT	2022.08.27	Szalonna					1	
GJ38	TUR PHI	2022.08.09	Tömörd	1				1	
BE15	LOC LUS	2022.07.12	Fenékpuszta	1					
BE18	LOC LUS	2022.07.13	Fenékpuszta	1					
BE27	LOC LUS	2022.07.23	Fenékpuszta	1					
BE45	ACR MEL	2022.08.15	Fenékpuszta	1					
BE47	HIR RUS	2022.08.20	Fenékpuszta		1				
BE49	HIR RUS	2022.08.15	Fenékpuszta		1				
BE56	HIR RUS	2022.08.23	Fenékpuszta		1				
BE57	HIR RUS	2022.08.23	Fenékpuszta		1				
BE59	NA	NA	Fenékpuszta		1				
BE61	HIR RUS	2022.08.20	Fenékpuszta		1				
BE101	RIP RIP	2022.08.27	Fenékpuszta		1				
BE149	HIR RUS	2022.09.01	Fenékpuszta		1				
BE150	HIR RUS	2022.09.01	Fenékpuszta		1				
BE151	HIR RUS	2022.09.01	Fenékpuszta		1				
BE160	HIR RUS	2022.09.03	Fenékpuszta		1				
BE164	PAN BIA	2022.09.05	Fenékpuszta				1		
BE176	ACR SCH	2022.09.23	Fenékpuszta			1			

ID	Bird Species	Date	Place	*Ornithomya avicularia*	*Onrnithomya biloba*	*Ornithomya fringillina*	*Ornithomya chloropus*	*Ornithoica turdi*	*Ornithoctona laticornis*
BE178	HIR RUS	2022.10.02	Fenékpuszta		1				
BE179	HIR RUS	2022.10.02	Fenékpuszta		2				
FB71	TUR MER	2015.06.17	Ócsa	1					
FB89	ACR SCI	2015.07.04	Ócsa	1					
FB93	ACR SCh	2015.07.04	Ócsa	1					
FB94	NA	2015.07.04	Ócsa	1					
FB102	TUR MER	2015.07.10	Ócsa	1					
FB105	TUR MER	2015.07.10	Ócsa	1					
FB108	TUR PHI	2015.07.10	Ócsa	1					
FB119	ASI OTU	2015.07.11	Ócsa	1					
FB158	ASI OTU	2015.07.15	Ócsa	2					
FB170	TUR MER	2015.07.16	Ócsa	1					
FB177	TUR MER	2015.07.16	Ócsa					1	
FB239	TUR MER	2015.07.27	Ócsa	1					
FB244	TUR MER	2015.07.28	Ócsa	1					
FB245	LOC LUS	2015.07.28	Ócsa	1					
FB283	NA	2015.08.06	Ócsa	1					
FB314	TUR MER	2015.08.23	Ócsa	1					
FB317	EMB SCH	2015.08.24	Ócsa					1	
FB355	ANT TRI	2015.09.08	Ócsa	2					
FB358	ANT TRI	2015.09.10	Ócsa	3					
FB421	ASI OTU	2015.10.23	Ócsa	1					
FB424	PAR CAE	2015.10.24	Ócsa	1					
FB439	REG REG	2015.10.30	Ócsa	1					
FB645	NA	2016.06.11	Ócsa	1					
FB656	ACR SCI	2016.07.02	Ócsa	1					
FB667	TUR MER	2016.07.10	Ócsa	1					
FB671	TUR MER	2016.07.10	Ócsa	2					
FB681	NA	2016.07.10	Ócsa	1					
FB686	TUR PHI	2016.07.11	Ócsa	2					
FB688	NA	2016.07.11	Ócsa			1			
FB703	LOC LUS	2016.07.15	Ócsa	1					
FB705	LOC LUS	2016.07.25	Ócsa	1					
FB772	LOC LUS	2016.08.08	Ócsa	1					
FB774	ACR MEL	2016.08.08	Ócsa	1					
FB859	LOC LUS	2016.09.09	Ócsa			1			
FB990	ASI OTU	2016.10.08	Ócsa	1					
FB993	PAR CAE	2016.10.09	Ócsa						1
FB1084	TUR MER	2017.05.26	Ócsa	1					
FB1086	TUR MER	2017.05.29	Ócsa	1					
FB1092	SYL ATR	2017.06.07	Ócsa	1					
FB1094	PIC VIR	2017.06.08	Ócsa	2					
FB1117	NA	2017.07.04	Ócsa	1					
FB1118	NA	2017.07.04	Ócsa	1					
FB1121	TUR PHI	2017.07.04	Ócsa	1					
FB1123	TUR MER	2017.07.04	Ócsa	1					
FB1125	ACR SCI	2017.07.04	Ócsa	1					

ID	Bird Species	Date	Place	*Ornithomya avicularia*	*Onrnithomya biloba*	*Ornithomya fringillina*	*Ornithomya chloropus*	*Ornithoica turdi*	*Ornithoctona laticornis*
FB1132	LOC LUS	2017.07.10	Ócsa	1					
FB1134	ACR SCh	2017.07.10	Ócsa	1					
FB1136	ACR ARU	2017.07.10	Ócsa	1					
FB1151	NA	2017.07.12	Ócsa	1					
FB1161	TUR MER	2017.07.15	Ócsa	1					
FB1166	TUR MER	2017.07.17	Ócsa					1	
FB1170	ACR SCI	2017.07.20	Ócsa	1					
FB1171	LOC LUS	2017.07.21	Ócsa	1					
FB1174	TUR MER	2017.07.21	Ócsa	1					
FB1175	LAN COL	2017.07.21	Ócsa					2	
FB1176	TUR MER	2017.07.22	Ócsa	1					
FB1187	ACR SCI	2017.07.27	Ócsa	1					
FB1200	ACR ARU	2017.07.29	Ócsa	1					
FB1214	TUR PHI	2017.08.03	Ócsa					1	
FB1227	LOC LUS	2017.08.10	Ócsa	1					
FB1232	TUR MER	2017.08.16	Ócsa					1	
FB1240	LOC LUS	2017.08.21	Ócsa					1	
FB1243	LOC LUS	2017.08.22	Ócsa	2					
FB1252	ACR MEL	2017.08.27	Ócsa			1			
FB1265	LAN COL	2017.09.02	Ócsa					1	
FB1266	EMB SCH	2017.09.02	Ócsa					1	
FB1274	ANT TRI	2017.09.10	Ócsa			1			
FB1278	HIR RUS	2017.09.11	Ócsa		1				
FB1285	HIR RUS	2017.09.26	Ócsa		1				
FB1286	HIR RUS	2017.09.26	Ócsa		1				
FB1287	SYL ATR	2017.09.28	Ócsa			1			
FB1328	PAR CAE	2017.10.19	Ócsa					1	
HS056	NA	2018.05.26	Ócsa	1					
HS058	SIT EUR	2018.05.29	Ócsa	1					
HS064	EMB SCH	2018.06.16	Ócsa	1					
HS065	NA	2018.06.16	Ócsa	1					
HS066	ACR SCI	2018.06.16	Ócsa	1					
HS069	SYL ATR	2018.06.17	Ócsa	1					
HS075	ACR SCH	2018.06.30	Ócsa	1					
HS077	ACR SCI	2018.06.30	Ócsa	1					
HS080	ACR SCI	2018.06.30	Ócsa	1					
HS081	TUR PHI	2018.07.01	Ócsa	1					
HS082	STR ALU	2018.07.01	Ócsa	1					
HS083	STR ALU	2018.07.01	Ócsa	4					
HS084	STR ALU	2018.07.01	Ócsa	2					
HS085	DEN MAJ	2018.07.01	Ócsa	1					
HS087	TUR PHI	2018.07.06	Ócsa	1					
HS093	ACR SCI	2018.07.08	Ócsa	1					
HS101	ACR SCI	2018.07.10	Ócsa	1					
HS105	ACR SCI	2018.07.10	Ócsa	1					
HS109	LOC LUS	2018.07.11	Ócsa	1					
HS116	LOC LUS	2018.07.13	Ócsa	1					

ID	Bird Species	Date	Place	*Ornithomya avicularia*	*Onrnithomya biloba*	*Ornithomya fringillina*	*Ornithomya chloropus*	*Ornithoica turdi*	*Ornithoctona laticornis*
HS117	ACR RIS	2018.07.13	Ócsa	1					
HS121	SYL ATR	2018.07.15	Ócsa					1	
HS127	LAN COL	2018.07.18	Ócsa					2	
HS128	DEN MAJ	2018.07.18	Ócsa	1					
HS133	ACR SCI	2018.07.20	Ócsa	1					
HS149	LAN COL	2018.07.26	Ócsa					1	
HS161	NA	2018.07.28	Ócsa	1					
HS183	LOC LUS	2018.08.05	Ócsa	1					
HS194	SYL ATR	2018.08.07	Ócsa	1					
HS221	ACR ARU	2018.08.17	Ócsa	1					
HS234	HIR RUS	2018.08.20	Ócsa		1				
HS242	ERI RUB	2018.08.21	Ócsa	1					
HS274	PRU MOD	2018.09.26	Ócsa			1			
HS288	PRU MOD	2018.10.09	Ócsa			1			
HS363	PAS MON	2019.05.19	Ócsa	1					
HS367	TUR MER	2019.05.31	Ócsa	1					
HS395	LUS MEG	2019.07.10	Ócsa	1					
HS397	LOC LUS	2019.07.10	Ócsa	2					
HS399	NA	2019.07.10	Ócsa	1					
HS400	LOC LUS	2019.07.11	Ócsa	1					
HS402	ACR SCI	2019.07.11	Ócsa	1					
HS403	LOC LUS	2019.07.11	Ócsa	3					
HS404	ACR ARU	2019.07.11	Ócsa	1					
HS407	NA	2019.07.12	Ócsa			1			
HS408	PHY COL	2019.07.14	Ócsa					1	
HS410	DEN MIN	2019.07.14	Ócsa	1					
HS411	LOC LUS	2019.07.14	Ócsa	1					
HS412	LOC LUS	2019.07.14	Ócsa	1					
HS415	ACR SCI	2019.07.15	Ócsa	1					
HS433	TUR PHI	2019.07.23	Ócsa	1					
HS434	ACR SCI	2019.07.23	Ócsa	1					
HS441	TUR PHI	2019.07.25	Ócsa	1					
HS449	ACR SCI	2019.08.03	Ócsa	1					
HS491	ANT TRI	2019.09.04	Ócsa			1			
HS492	ANT TRI	2019.09.04	Ócsa			1			
HS493	HIR RUS	2019.09.04	Ócsa		1				
HS494	HIR RUS	2019.09.05	Ócsa		1				
SH011	SYL ATR	2019.09.16	Ócsa			1			
SH021	ERI RUB	2019.10.02	Ócsa	1					
SH029	NA	NA	Ócsa				1		
SH040	PAR MAJ	2019.11.23	Ócsa			1			
SH066	DEN MAJ	2020.05.30	Ócsa	3					
SH068	NA	2020.05.30	Ócsa	1					
SH069	NA	2020.06.06	Ócsa	1					
SH070	PAR MAJ	2020.06.06	Ócsa	1					
SH076	TUR MER	2020.06.06	Ócsa	2					
SH083	SYL ATR	2020.06.27	Ócsa	1					

ID	Bird Species	Date	Place	*Ornithomya avicularia*	*Onrnithomya biloba*	*Ornithomya fringillina*	*Ornithomya chloropus*	*Ornithoica turdi*	*Ornithoctona laticornis*
SH086	LUS MEG	2020.06.27	Ócsa	1					
SH089	LOC LUS	2020.07.10	Ócsa	1					
SH090	ACR SCI	2020.07.10	Ócsa	1					
SH092	LOC LUS	2020.07.10	Ócsa	2					
SH100	LOC LUS	2020.07.10	Ócsa	1					
SH120	ACR SCH	2020.07.12	Ócsa	1					
SH150	TUR MER	2020.07.28	Ócsa	1					
SH151	TUR MER	2020.08.01	Ócsa	1					
SH158	ACR ARU	2020.08.08	Ócsa	1					
SH164	TUR MER	2020.07.30	Ócsa	1					
SH165	LOC LUS	2020.07.30	Ócsa	2					
SH175	TUR MER	2020.08.27	Ócsa	1					
SH190	HIR RUS	2020.09.05	Ócsa		1				
SH192	HIR RUS	2020.09.05	Ócsa	1					
SH193	HIR RUS	2020.09.05	Ócsa		1				
OC11	ACR ARU	2020.09.19	Ócsa	1					
OC23	TUR PHI	2020.10.05	Ócsa	1					
OC34	PRU MOD	2020.10.31	Ócsa				1		
OC108	NA	2021.07.24	Ócsa	1					
OC150	LOC LUS	2021.08.14	Ócsa	2					
OC161	ACR ARU	2021.08.26	Ócsa	1					
OC168	ACR SCI	2021.08.28	Ócsa			1			
OC175	SYL COM	2021.09.04	Ócsa			1			
OC182	SYL ATR	2021.09.09	Ócsa			1			
Total				168	23	17	3	25	1
