# Supplementary material for: Contributions to our knowledge on avian louse flies (Hippoboscidae: Ornithomyinae) with the first European record of the African species Ornithoctona laticornis
Source: Parasit Vectors. 2024 May 27;17:237. doi: 10.1186/s13071-024-06303-8 (PMC11129389; doi:10.1186/s13071-024-06303-8)
Supplement: Supplementary file 2 — Additional file 2 Table S2. Categorization of the bird species found to be louse fly infested during the study, according to their migration habit, habitat and feeding place. [file 13071_2024_6303_MOESM2_ESM.pdf]

Table S2: Categorization of the bird species found to be louse fly infested during the study, according to their migration habit, habitat and feeding place

HURING code	Scientific name	English name	Migration habit	Habitat	Feeding place
ACR ARU	*Acrocephalus arundinaceus*	Great Reed Warbler	LDM	R	A
ACR MEL	*Acrocephalus melanopogon*	Moustached warbler	SDM	R	A
ACR RIS	*Acrocephalus palustris*	Marsh Warbler	LDM	R	G
ACR SCH	*Acrocephalus schoenobaenus*	Sedge Warbler	LDM	R	A
ACR SCI	*Acrocephalus scirpaceus*	Eurasian Reed Warbler	LDM	R	A
ANT TRI	*Anthus trivialis*	Tree Pipit	LDM	M/F	G/A
ASI OTU	*Asio otus*	Long-eared Owl	R/SDM	F	G
COR NIX	*Corvus cornix*	Hooded Crow	R	M	G
DEN MAJ	*Dendrocopos major*	Great Spotted Woodpecker	R/SDM	F	A
DEN MIN	*Dendrocopos minor*	Lesser Spotted Woodpecker	R	F	A
EMB CIT	*Emberiza citrinella*	Yellowhammer	R/SDM	M	G
EMB SCH	*Emberiza schoeniclus*	Common Reed Bunting	R/SDM	R	A
ERI RUB	*Erithacus rubecula*	European Robin	R/SDM	F	G
HIR RUS	*Hirundo rustica*	Barn Swallow	LDM	M	only flying insects
LAN COL	*Lanius collurio*	Red-backed Shrike	LDM	M	G
LOC LUS	*Locustella luscinioides*	Savi's Warbler	LDM	R	A
LUS MEG	*Luscinia megarhynchos*	Common Nightingale	LDM	F	G
PAN BIA	*Panurus biarmicus*	Bearded Reedling	R/SDM	R	A
PAR CAE	*Cyanistes caeruleus*	Eurasian Blue Tit	R	F	A
PAR MAJ	*Parus major*	Great Tit	R	F	A
PAS MON	*Passer montanus*	Eurasian Tree Sparrow	R	M	G
PHY COL	*Phylloscopus collybita*	Common Chiffchaff	SDM	F	A
PIC VIR	*Picus viridis*	European Green Woodpecker	R	F	A
PRU MOD	*Prunella modularis*	Dunnock	SDM	F	A
REG REG	*Regulus regulus*	Goldcrest	R/SDM	F	A
RIP RIP	*Riparia riparia*	Sand Martin	LDM	sand walls	only flying insects
SIT EUR	*Sitta europaea*	Eurasian Nuthatch	R	F	A
STR ALU	*Strix aluco*	Tawny Owl	R/SDM	M/F	G/A
SYL ATR	*Sylvia atricapilla*	Eurasian Blackcap	SDM	F	A
SYL COM	*Sylvia communis*	Common Whitethroat	LDM	M	G/A
TUR MER	*Turdus merula*	Common Blackbird	R/SDM	M/F	G
TUR PHI	*Turdus philomelos*	Song Thrush	SDM	F	G

Abbreviations: **Migration habit:** R: residedent; SDM: short-distance migrant; LDM: long-distance migrant
Habitat: R: reed; M: meadow; F: forest
Feeding place: G: ground; A: above ground
